# Genes Required for the Anti-fungal Activity of a Bacterial Endophyte Isolated from a Corn Landrace Grown Continuously by Subsistence Farmers Since 1000 BC

**DOI:** 10.3389/fmicb.2016.01548

**Published:** 2016-10-04

**Authors:** Hanan R. Shehata, Cassandra L. Ettinger, Jonathan A. Eisen, Manish N. Raizada

**Affiliations:** ^1^Department of Plant Agriculture, University of Guelph, GuelphON, Canada; ^2^Department of Microbiology, School of Pharmacy, Mansoura UniversityMansoura, Egypt; ^3^Genome Center, University of California Davis, DavisCA, USA; ^4^Department of Evolution and Ecology, University of California Davis, DavisCA, USA; ^5^Department of Medical Microbiology and Immunology, University of California Davis, DavisCA, USA

**Keywords:** *Sclerotinia homoeocarpa*, *Zea*, Chapalote, endophyte, c-di-GMP, YajQ, antifungal, *Burkholderia gladioli*

## Abstract

Endophytes are microbes that inhabit internal plant tissues without causing disease. Some endophytes are known to combat pathogens. The corn (maize) landrace Chapalote has been grown continuously by subsistence farmers in the Americas since 1000 BC, without the use of fungicides, and the crop remains highly valued by farmers, in part for its natural tolerance to pests. We hypothesized that the pathogen tolerance of Chapalote may, in part, be due to assistance from its endophytes. We previously identified a bacterial endophyte from Chapalote seeds, *Burkholderia gladioli* strain 3A12, for its ability to combat a diversity of crop pathogens, including *Sclerotinia homoeocarpa*, the most important fungal disease of creeping bentgrass, a relative of maize used here as a model system. Strain 3A12 represents a unique opportunity to understand the anti-fungal activities of an endophyte associated with a crop variety grown by subsistence farmers since ancient times. Here, microscopy combined with Tn5-mutagenesis demonstrates that the anti-fungal mode of action of 3A12 involves flagella-dependent swarming toward its pathogen target, attachment and biofilm-mediated microcolony formation. The mutant screen revealed that YajQ, a receptor for the secondary messenger c-di-GMP, is a critical signaling system that mediates this endophytic mobility-based defense for its host. Microbes from the traditional seeds of farmers may represent a new frontier in elucidating host–microbe mutualistic interactions.

## Introduction

Fungal diseases are widespread among all plant species and represent persistent threats to global agriculture (Anderson et al., 2004; Fisher et al., 2012). One means of combating fungal pathogens involves mutualistic interactions with endophytes, defined as microbes that inhabit the internal living tissues of plants without showing disease symptoms (Hallmann et al., 1997; Strobel, 2003; Johnston-Monje and Raizada, 2011b). Endophytes control plant pathogens through direct antagonism (production of antimicrobial compounds), competition for nutrients or space, and/or induction of host plant defense mechanisms (Reinhold-Hurek and Hurek, 2011; Mousa and Raizada, 2015). Worldwide, subsistence farmers often prefer to grow traditional crop varieties (landraces), typically without the use of synthetic fungicides (Goron and Raizada, 2015; Thilakarathna and Raizada, 2015). We hypothesize that some of these landraces possess natural tolerance to pathogens, enabled by endophytes with anti-fungal genes. In particular, landraces that have been maintained by farmers over 100s or 1000s of years may be especially robust and worth investigating as unique sources for mutualistic endophytes.

We previously isolated a bacterial endophyte (strain 3A12) from seeds of a traditional landrace of maize (corn, *Zea mays* ssp. mays) from Mexico known as Chapalote (Johnston-Monje and Raizada, 2011a). The draft genome sequence of 3A12 identified it as a novel strain of *Burkholderia gladioli* with a genome size of ∼8.5 Mbp (Ettinger et al., 2015).

Chapalote has an ancient origin and is considered a missing link between wild and modern maize (Wessel et al., 2013). Chapalote emerged in the archaeological records > 3000 years ago in Mexico (Wellhausen, 1952). Paleogenomics suggests that it was one of the first maize races to enter the USA > 2000 years ago (da Fonseca et al., 2015). Archaeological remains of Chapalote have been found in the Boca Negra Cave (25 BC) and Site BR-45 (AD 370) near Albuquerque, New Mexico (Sturtevant, 1978). The landrace is still grown today in Mexico by traditional farmers (Bonavia, 2013), suggesting that it remains highly valued.

*Burkholderia gladioli* strain 3A12 was initially identified in a screen for endophytes (Shehata et al., 2016) with ability to suppress the fungal pathogen *Sclerotinia homoeocarpa*, which infects grass relatives of maize including creeping bentgrass (*Agrostis stolonifera*; Rioux et al., 2014) which we initiated as a model system. Subsequently, strain 3A12 was shown to suppress the *in vitro* growth of 16 out of 18 diverse crop fungi that were tested, suggesting that it has a wide target spectrum (Shehata et al., 2016). Strain 3A12 represents a unique opportunity to investigate the anti-fungal activity of an endophyte that was isolated from a crop genotype grown continuously by subsistence farmers since antiquity. Previous studies demonstrated that the mechanisms of antifungal activity in the genus *Burkholderia*, include the production of a volatile organic compound (cyclic terpene), and antimicrobials such as phenazine, chitinase, lipopeptides, quinolinones, altericidins, pyrrolnitrin, cepacidines, siderophores, and lipopeptides (Burkhead et al., 1994; Cartwright et al., 1995; Moon et al., 1996; Kang et al., 1998; Kong et al., 2001; Vial et al., 2007; Schmidt et al., 2009; Elshafie et al., 2012).

## Materials and Methods

### Sources of Biological Materials

The bacterial endophyte 3A12 (GenBank: JRGO00000000, strain UCD-UG CHAPALOTE) was isolated from seeds of the Mexican maize landrace Chapalote (Johnston-Monje and Raizada, 2011a). The *S. homoeocarpa* strain was obtained from the Guelph Turfgrass Institute, Guelph, Canada. Creeping bentgrass (CB) (*Agrostis stolonifera*) PENN A-4 seeds were obtained from the Ontario Seed Company, Kitchener, ON, Canada.

### Testing Candidate Mutants for Loss of Antifungal Activity on Creeping Bentgrass

Bacteria were applied as seed coats onto surface sterilized creeping bentgrass seeds grown in glass tubes. After 10 days of growth, the tubes were inoculated with disks of *S. homoeocarpa*. Please see Supplementary Materials and Methods.

### GFP-Tagging and Microscopic Imaging of Strain 3A12

Competent cells of strain 3A12 were GFP tagged using plasmid vector (pDSK-GFPuv; Wang et al., 2007). GFP-tagged strain 3A12 was introduced onto creeping bentgrass seeds as a seed coat. Plants were examined under a confocal laser scanning microscope (SP5, Leica). Please see Supplementary Materials and Methods.

### Scanning Electron Microscopy (SEM) of Endophytes

Scanning electron microscopy (SEM) was used to visualize the endophytes (Hayat, 1989). The endophytes were plated on PDA plates. After 24 h of incubation at 37°C, bacterial colonies were scraped and suspended in phosphate buffer (pH 7), centrifuged, washed twice by resuspension and centrifugation, and finally the cultures were resuspended in the same buffer. One drop of each suspension was transferred onto a carbon disk and let dry for 1 h. The resulting dried bacteria were washed in phosphate buffer, fixed by 2% glutaraldehyde for 1 h, treated with 1% osmium tetroxide for 30 min then gradually dehydrated using an ethanol series (50, 70, 80, 90, and 100%). The resulting bacterial films were coated with gold and examined under SEM (Hitachi S-570 SEM, Hitachi High Technologies, Tokyo, Japan) at the Imaging Facility, Department of Food Science, University of Guelph, Canada.

### Characterization of *In vitro* Interactions between Endophyte 3A12 and *S. homoeocarpa*

The *in vitro* interaction between endophyte 3A12 and the fungal pathogen *S. homoeocarpa* was examined on a microscope slide. *S. homoeocarpa* was cultured in YPD media for 3 days at 25°C at 80 rpm. Endophyte 3A12 was cultured in LB liquid medium overnight at 37°C with shaking at 250 rpm.

Five hundred microliters of PDA was spread on a previously sterilized glass slide placed in a Petri dish. After solidification, a fragment of *S. homoeocarpa* mycelia was transferred to the center of the slide, and then 20 μl of 3A12 culture was transferred to one side of *S. homoeocarpa* mycelia, and on the other side 20 μl of LB media was applied. Petri dishes containing the slides were incubated at 25°C overnight. Slides were stained using Neutral Red (#N6264, Sigma, USA) or Evans blue (#206334, Sigma, USA), then examined under a light microscope (B1372, Axiophot, Zeiss, Germany) using Northern Eclipse software.

### Identification of Gene(s) Required for the Antifungal Activity

The EZ-Tn5 < R6Kγori/KAN-2 > Tnp Transposome^TM^ Kit was used (TSM08KR, Epicentre, USA) to create random mutations in strain 3A12, then ∼3000 mutants were tested for loss of antifungal activity. Candidate mutants were characterized using plasmid rescue and BLAST analysis. Insertions were confirmed using genetic complementation. Please see Supplementary Materials and Methods.

### Biofilm Formation Assay

Wild type 3A12 and candidate mutants were cultured in LB (supplemented with 25 μg/ml kanamycin in case of mutants) overnight at 37°C and 250 rpm. The OD_595_ for all cultures were adjusted to 1.0. Cultures were diluted 1:100 in LB, then 200 μl from each diluted culture were transferred to a well in a microtiter plate (3370, Corning Life Sciences, USA) in 4–5 replicates. The negative control wells received LB only. The microtiter plate was covered with its lid, and incubated for 2 days at 37°C. The plate was emptied by aspiration and washed three times with shaking in sterile saline to remove Planktonic bacterial cells. Adherent cells were fixed with 200 μl of 99% methanol for 15 min. The plate was left to air dry, then 200 μl of 2% crystal violet (94448, Sigma) were added to each well and left to stain cells for 5 min. The plate was washed with water, then left to air dry. The crystal violet was solubilized in 160 μl of 33% (v/v) glacial acetic acid. The plate was read by a spectrophotometer at 570 nm (Stepanović et al., 2000). The entire experiment was repeated independently.

### Bacterial Swarming Motility Assay

Semisolid LB agar (0.3% agar W/V) was prepared (Easom and Clarke, 2008), and 40 ml were transferred into Petri dishes (150 mm diameter). Wild type 3A12 and candidate mutants were cultured in LB (supplemented with 25 μg/ml kanamycin in the case of mutants) overnight at 37°C and shaking at 250 rpm. The OD_595_ for all cultures were adjusted to 1.0, and then 15 μl from each culture were spotted on each Petri dish. Plates were incubated at 30°C, and motility was measured as the resulting diameter of the colony. Each culture was tested as seven colonies in triplicate plates (*n* = 21). The entire experiment was repeated independently.

### Transmission Electron Microscopy (TEM)

Wild type 3A12 and candidate mutants were cultured in LB (supplemented with 25 μg/ml kanamycin in the case of mutants) overnight at 37°C with shaking at 250 rpm. From each culture, 5 μl were pipetted onto a 200-mesh copper grid coated with formvar and carbon. The excess fluid was removed onto a filter, and the grid was stained for 10 s with 2% uranyl acetate. The resulting sample was viewed in the FEI Tecnai F20 G2 operating at 200 kV. Images were collected using the Gatan 4K CCD camera and the Digital Micrograph software. Imaging was carried out at the Electron Microscopy Unit, University of Guelph.

### Examination of Swarming and Colony Formation around the Fungal Target

Wild type 3A12 and mutants were examined for swarming motility and colony formation around *S. homoeocarpa* on a microscope slide. Endophyte 3A12 and mutants were cultured in LB liquid medium overnight at 37°C with shaking at 250 rpm. Simultaneously, *S. homoeocarpa* was cultured for 3 days in YPD media at 25°C with shaking at 80 rpm. Glass slides were autoclaved, placed in Petri dishes, then 0.5 ml of PDA was spread on each glass slide. A fragment of *S. homoeocarpa* mycelia was transferred to the center of each slide, the Petri dishes were incubated overnight at 25°C, then 10 μl of each culture were transferred to the sides of *S. homoeocarpa* mycelia on 2–3 glass slides and incubated at 25°C for 5 h. Slides were examined under a light microscope (B1372, Axiophot, Zeiss, Germany) using Northern Eclipse software.

### Chitinase Activity Assay

In an initial experiment, chitinase activity in wild type strain 3A12 was measured using the Chitinase Assay Kit (CS0980, Sigma) according to manufacturer recommendations. 4-nitrophenyl *N,N*′-diacetyl-β-D-chitobioside and 4-nitrophenyl *N*-acetyl-β-D-glucosaminide were used as enzyme substrates. Endophyte 3A12 was cultured in LB media for 2 days at 37°C and shaking at 250 rpm. Ten microliters of the 3A12 culture (OD_595_ = 0.8) or the chitinase control enzyme were used. The blank treatment consisted of 90 μl of the substrate with 10 μl of LB media. To measure whether chitinase activity is induced by *S. homoeocarpa*, 3A12 was cultured with and without the fungus, then chitinase activity was measured using 4-nitrophenyl *N*-acetyl-β-D-glucosaminide as a substrate. The blank treatment consisted of 90 μl of the substrate with 10 μl of LB media (with and without the fungus). Each assay was performed in triplicate, and each experiment was replicated. In a second experiment, chitinase activity was measured in the candidate mutants in triplicate using 4-nitrophenyl *N*-acetyl-β-D-glucosaminide as a substrate and compared to wild type, following the same protocol as noted above. For both experiments, the chitinase activity was calculated according to the following formula:

(1) $$\begin{array}{c} Units/ml\;=\;\frac{(A_{405}\mathrm{sample}-A_{405}\mathrm{blank})\;\times 0.05\times 0.3\times 20}{(A_{405}\;\mathrm{standard}\;\times 30\;\times \;0.01).} \end{array}$$

### Statistical and Graphical Analysis

Prism 6 (GraphPad Software, USA) was used for graphical displays and statistical analysis (Student’s *t*-test).

## Results

### Microscopic Imaging of Strain 3A12

**FIGURE 1:**
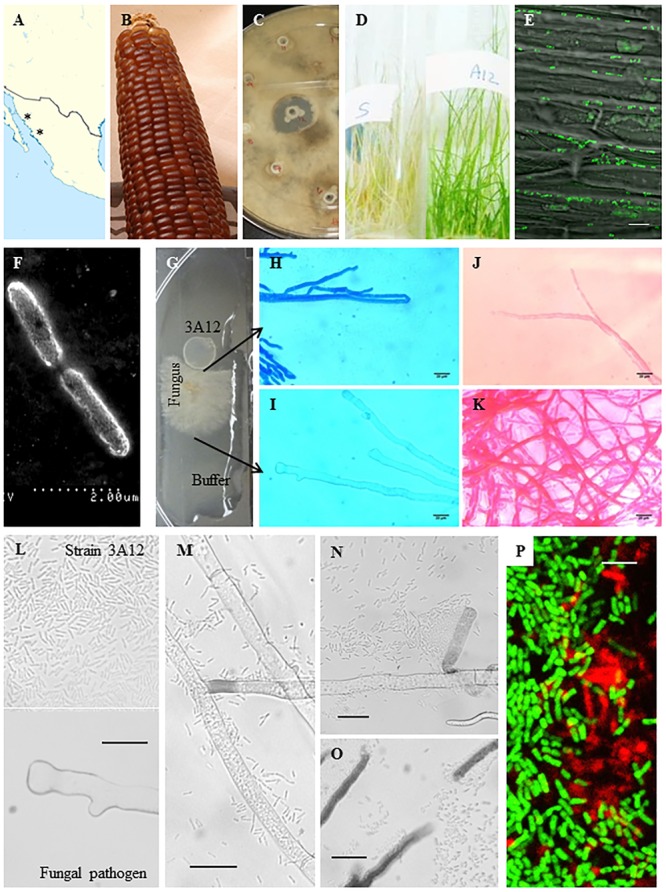
**Characterization of antifungal strain 3A12. (A)** Map showing geographical origin (asterisks) of the ancient maize landrace Chapalote (the source of endophyte 3A12). **(B)** Picture of Chapalote plants. **(C)** *In vitro* antifungal activity of strain 3A12 against *S. homoeocarpa*. **(D)** *In planta* antifungal activity of strain 3A12 (right) against *S. homoeocarpa* disease (dollar spot) compared to non-inoculated plants (left). **(E)** Visualization of GFP tagged strain 3A12 in creeping bentgrass shoots using confocal microscopy following seed coating. The scale bar is 10 μm. **(F)** Scanning electron microscopy image confirming the rod shape of strain 3A12. The scale bar is 2 μm. **(G–O)** The interaction of strain 3A12 with *S. homoeocarpa* **(G)** on a microscope slide with agar, showing *S. homoeocarpa* mycelia **(H,J)** in contact with strain 3A12, or **(I,K)** the buffer control, after 24 h of incubation followed by staining the mycelia with **(H,I)** Evans blue (death stain) or **(J,K)** Neutral red (vitality stain). The scale bars in **(H–K)** are 20 μm. **(L–O)** Time course of the interaction between strain 3A12 with *S. homoeocarpa* at **(L)** time 0, **(M)** after 5 h of incubation, **(N)** after 7 h of incubation, and **(O)** after 24 h of incubation where strain 3A12 breaks fungal mycelia. **(P)** Confocal microscopy image showing biofilm formation surrounding GFP tagged wild type 3A12 endophytic cells on a microscope slide stained with a red biofilm fluorescent stain (FilmTracer^TM^ SYPRO^®^ Ruby Biofilm Matrix Stain). The map image **(A)** is licensed under the Creative Commons Attribution-Share Alike 3.0 Unported license. The picture of Chapalote corn **(B)** is courtesy of CIMMYT under the Creative Commons BY-NC-SA 2.0 license.

Following its isolation from maize landrace Chapalote (**Figures 1A,B**), endophyte 3A12 showed anti-fungal activity against *S. homoeocarpa in vitro* (**Figure 1C**) and in creeping bentgrass (**Figure 1D**). GFP-tagged 3A12 was applied as a seed coat and as a spray onto creeping bentgrass seeds. Examining creeping bentgrass plants under a confocal microscope showed that strain 3A12 colonized both roots and shoots confirming its endophytic ability in creeping bentgrass (**Figure 1E**, **Supplementary Figure S3** and data not shown). The strain was also examined using a scanning electron microscope (SEM) which revealed a rod shape phenotype (**Figure 1F**) consistent with its classification as *B. gladioli* (Ettinger et al., 2015).

### *In vitro* Interactions between Endophyte 3A12 and *S. homoeocarpa*

Growing endophyte 3A12 side by side with *S. homoeocarpa* on a microscope slide (**Figure 1G**) then staining with Evans blue (which stains dead tissue as blue), revealed that the mycelia stained blue only when in close contact with 3A12 and not at the control LB side (**Figures 1H,I**). Staining with neutral red (which stains live tissue as red) showed that mycelia were stained only on the control LB side while faint staining was observed when in contact with strain 3A12 (**Figures 1J,K**). These results suggest that strain 3A12 has fungicidal activity, rather than fungistatic activity, against *S. homoeocarpa.* When wild type strain 3A12 was spotted at a distance from *S. homoeocarpa* mycelia (**Figure 1L**), bacteria were observed to swarm, adhere to and form microcolonies around its fungal target (**Figures 1M,N**). Mycelia cleavage was observed associated with 3A12 microcolonies (**Figure 1O**), confirming the fungicidal activity of 3A12. Staining 3A12 cells with a biofilm detection stain (FilmTracer^TM^ SYPRO^®^ Ruby Biofilm Matrix Stain) demonstrated that 3A12 microcolony formation was mediated by biofilm (red; **Figure 1P**).

### Identification of Genes Required for the Fungicidal Activity

**FIGURE 2:**
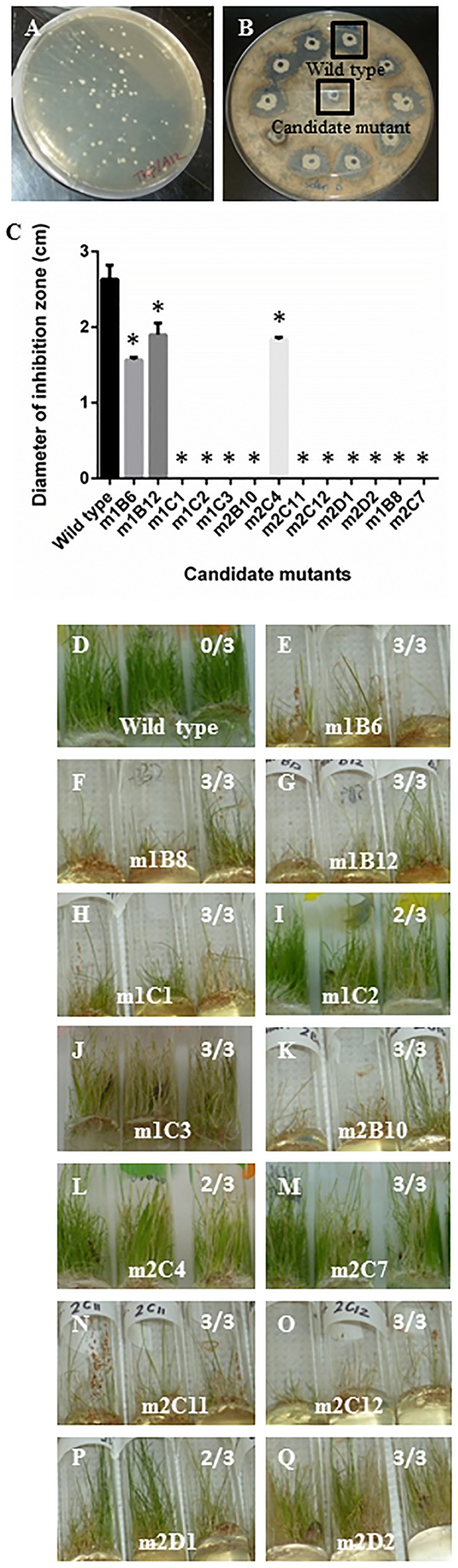
**Screen for endophyte strain 3A12 mutants that show loss or reduction in anti-fungal activity. (A)** *Tn5* insertion strains growing on LB-kanamycin agar. **(B)** Example PDA agar plate containing *S. homoeocarpa* pathogen showing loss of inhibition zones associated with a candidate mutant compared to wild type 3A12. **(C)** Summary of candidate 3A12 mutants showing lost or reduced inhibition zones of *S. homoeocarpa* growth. Asterisks indicate significant difference in mean diameter of the inhibition zone compared to the wild type strain. **(D–Q)** *In planta* confirmation of loss of antifungal activity in creeping bentgrass following seed coating with each endophyte strain as indicated. The numbers shown indicate the number of tubes with sick plants out of three tubes.

To identify the endophytic genes required for its fungicidal activity, ∼3000 independent *Tn5* mutants were screened *in vitro* (in triplicate) for loss of antifungal activity against *S. homoeocarpa* (**Figures 2A,B**). This screen resulted in 13 candidate insertions (mutants) that showed loss or reduction in the diameter of inhibition zones of *S. homoeocarpa* growth (**Figure 2C**). The 13 candidate mutations were confirmed to lose the plant protective activities (**Figures 2D–Q**), though m1C2, m2C4, m2D1 demonstrated incomplete loss of anti-fungal activity (2/3 tubes, **Figure 2O**). The sequences flanking the *Tn5* insertions for 10 of the 13 candidate genes were successfully identified following plasmid rescue using the reference genome sequence of strain 3A12 (Supplementary Table S1; Ettinger et al., 2015). The retrieved gene sequences were further analyzed using BLASTN searches against GenBank. Some genes were isolated multiple times, narrowing the list to five unique genes required for anti-fungal activity (**Table 1**). The genes were predicted to encode: (1) a YajQ ortholog, (2) a fatty acid desaturase (Fad), (3) a gene within a Tol operon (*ybgC* ortholog, 4-hydroxybenzoyl-CoA thioesterase), (4) a hypothetical protein containing a lysine-tRNA synthetase domain (Lys) (identified using the Conserved Domain Database), and (5) arginine/ornithine/lysine decarboxylase (Adc). The corresponding, predicted wild type sequences were amplified (Supplementary Table S2) and used to complement four of the mutants [*ybgC(tol)*-2C11::Tn5, *yajQ*-1B12::Tn5, *adc*-2D2::Tn5 and *fad*-1C1::Tn5] (**Supplementary Figure S1**) while the mutation in the lysine-tRNA synthetase like gene was isolated independently three times (*lys*-1B6::Tn5, *lys*-1C3::Tn5, *lys*-2D1::Tn5). Candidate mutants were tested for their growth rates (OD_595 nm_) to exclude the possibility that they lost their antifungal activity because of a slower growth rate; none were significantly different (Supplementary Table S3).

**Table 1:** Summary of the genes^#^ which are essential for the antifungal activity of strain 3A12 against *Sclerotinia homoeocarpa.*

Short mutant name	Full mutant name	BLAST against 3A12 genome sequence		BLAST against GenBank		
		Scaffold position	Identity	E-value	Identity	E-value
*m1B12*	*yajQ*-1B12::Tn5	6.1	YajQ protein	0	^∗^YajQ protein	^∗^5e-44
		115014				
		-114529				
*m1C1*	*fad*-1C1::Tn5	6.1	Fatty acid desaturase (EC	0	Fatty acid desaturase	0
		174150	1.14.19.1); Delta-9 fatty acid		family protein	
*m2C12*	*fad*-2C12::Tn5	-175334	desaturase (EC 1.14.19.1)			
*m1B6*	*lys*-1B6::Tn5	34.1	Hypothetical protein	e-108	^∗∗^lysine-tRNA	^∗∗^6.2e-03
*m1C3*	*lys*-1C3::Tn5	-89251			synthetase	
*m2D1*	*lys*-2D1::Tn5	89057				
*m2B10*	*ybgC(tol)-*2B10::Tn5	15.1	4-hydroxybenzoyl-CoA	0	4-hydroxybenzoyl-CoA	0
*m2C4*	*ybgC(tol)-*2C4::Tn5	99586	thioesterase family active site		thioesterase	
*m2C11*	*ybgC(tol)-*2C11::Tn5	-100056	(Ton and Tol operon)			
*m2D2*	*adc*-2D2::Tn5	75.1	Arginine decarboxylase (EC	0	Orn/Lys/Arg	0
		20314	4.1.1.19); Ornithine		decarboxylase, N-	
		-22593	decarboxylase (EC 4.1.1.17);		terminal domain protein	
			Lysine decarboxylase (EC			
			4.1.1.18)			

*#Identifiable sequences associated with mutants m1C2, m1B8, and m2C7 could not be retrieved.*

*^∗^BLASTX.*

*^∗∗^Conserved Domain Database.*

### Effect of Mutants on Virulence Phenotypes

The mutants were phenotyped for disruptions in the virulence phenotypes (biofilm formation, motility, attachment) associated with the anti-fungal activity of wild type 3A12 (Supplementary Table S3).

#### Biofilm Formation

**FIGURE 3:**
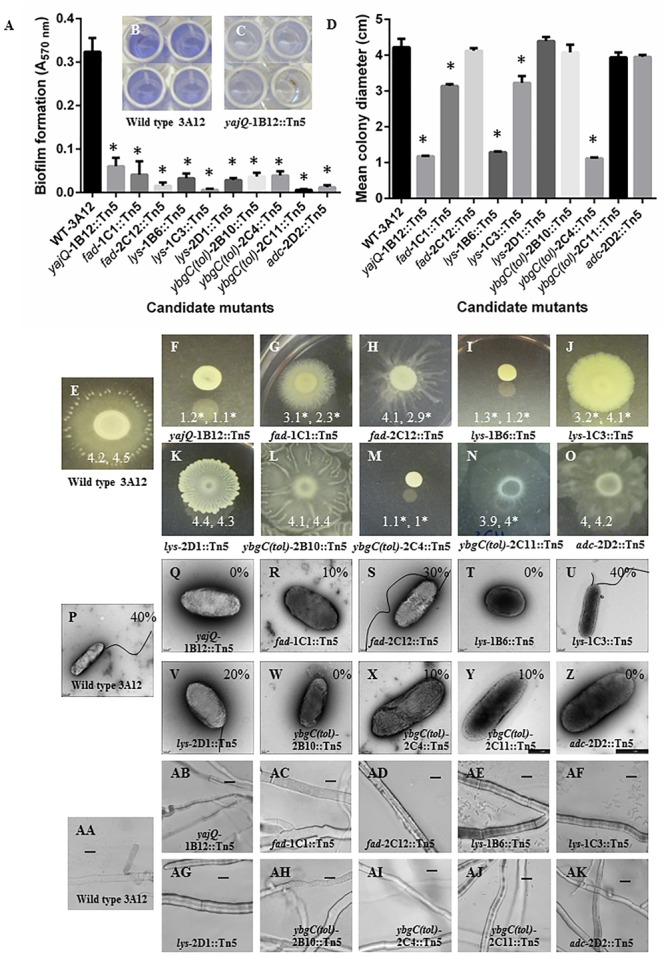
**Characterization of the candidate anti-fungal mutants of endophyte strain 3A12. (A–C)** Colorimetric biofilm formation assays. **(A)** Graph showing biofilm formation (Crystal violet, A_570 nm_) for the wild type and candidate mutants. **(B,C)** Representative images for **(B)** wild type strain 3A12, and **(C)** mutant *yajQ*-1B12::Tn5. **(D–O)** Motility assay of the candidate anti-fungal mutants on LB agar plates. **(D)** Graph showing mean colony diameter (cm) for the wild type and candidate mutants (*n* = 21). Asterisks indicate significant difference from wild type strain 3A12. **(E–O)** Representative images of colony formation for wild type 3A12 and candidate mutants. The two numbers shown in **(E–O)** indicate the mean colony diameter (*n* = 21) with the asterisks indicating significant difference in two independent trials. **(P–Z)** Detection of flagella using transmission electron microscopy (TEM) for wild type strain 3A12 and mutants. The numbers shown in **(P–Z)** indicate the percentage of bacteria that were observed to have flagella. **(AA–AK)** Examination of mutants for their ability to swarm, attach and form microcolonies around their fungal target. Shown are light microscopy images of *S. homoeocarpa* mycelia grown on a microscope slide side by side with wild type 3A12 or mutant strains. Bacteria are not visible around the mycelia in some images which reflects the inability of those strains to reach the fungal pathogen. The scale bar is 20 μm.

The mutants were assayed for alterations in biofilm formation by quantifying cells that bind to 96-well plates following staining with crystal violet which absorbs at 570 nm (Stepanović et al., 2000). Wild type strain 3A12 showed intense crystal violet staining suggestive of biofilm formation (**Figures 3A,B**). By contrast, the absorbance A_570 nm_ readings from all 10 candidate mutants were significantly different than those of strain 3A12 (**Figures 3A–C** and Supplementary Table S3). These results suggest that all the candidate mutants have impaired biofilm formation compared to wild type.

#### Motility

The motility of candidate mutants was quantified by measuring the diameters of colonies on low percentage agar plates (Easom and Clarke, 2008). The colony diameters of mutants *lys*-1B6::Tn5, *lys*-1C3::Tn5, *yajQ*-1B12::Tn5, *fad*-1C1::Tn5, and *ybgC(tol)*-2C4::Tn5 were significantly different from that of wild type strain 3A12 in two independent trials. Mutants *fad*-2C12::Tn5 and *ybgC(tol)*-2C11::Tn5 were significantly different in one trial only while colony diameters of mutants *adc*-2D2::Tn5, *lys*-2D1::Tn5 and *ybgC(tol)-*2B10::Tn5 were not significantly different in either independent trial (**Figures 3D–O**; Supplementary Table S3).

#### Flagella Number

Examining wild type 3A12 using transmission electron microscopy (TEM) showed that flagella could be detected in 40% of examined cells. Mutants *fad*-1C1::Tn5, *fad*-2C12::Tn5, *ybgC(tol)*-2C11::Tn5, *ybgC(tol)*-2C4::Tn5, and *lys*-2D1::Tn5 also possessed flagella in 10–40% of examined cells, as indicated, while mutants *ybgC(tol)-*2B10::Tn5, *lys*-1B6::Tn5, *yajQ*-1B12::Tn5, and *adc*-2D2::Tn5 were found to have no flagella on any of the observed cells (*n* = 10–15; **Figures 3P–Z**; Supplementary Table S3).

#### Swarming, Adhesion, and Colony Formation

In contrast to the swarming, adherence and microcolony formation behavior of wild type strain 3A12 around its fungal target (**Figure 3AA**), the majority of the mutants [*yajQ*-1B12::Tn5, *fad*-1C1::Tn5, *fad*-2C12::Tn5, *ybgC(tol)-*2B10::Tn5, *ybgC(tol)*-2C4::Tn5, *ybgC(tol)*-2C11::Tn5, and *adc*-2D2::Tn5] showed loss of these traits, while mutants *lys*-1B6::Tn5, *lys*-1C3::Tn5 and *lys*-2D1::Tn5 were still able to swarm and adhere to the target fungus but showed reduced microcolony formation (**Figures 3AB–AK**).

### Additional Gene Predictions and Assay for Chitinase Activity

RAST-server based bioinformatic mining of the 3A12 genome (Ettinger et al., 2015) revealed the presence of several additional candidate anti-fungal genes including chitinase (**Supplementary Figure S2A**). A few of these genes were present in the genome as multiple copies, perhaps causing genetic redundancy. Strain 3A12 was confirmed to have chitinase activity using two different enzyme substrates, 4-nitrophenyl *N,N*′-diacetyl-β-D-chitobioside and 4-nitrophenyl *N*-acetyl-β-D-glucosaminide (**Supplementary Figure S2B**). Strain 3A12 was tested for chitinase activity in the presence of *S. homoeocarpa*, using 4-nitrophenyl *N*-acetyl-β-D-glucosaminide as a substrate. The chitinase activity was induced by *S. homoeocarpa* (**Supplementary Figure S2c**). Chitinase activity was measured in the candidate mutants using 4-nitrophenyl *N*-acetyl-β-D-glucosaminide as a substrate. When compared to the chitinase activity of wild type strain 3A12, mutant *yajQ*-1B12::Tn5 showed clearly reduced chitinase activity compared to wild type (Supplementary Table S3).

## Discussion

### Endophyte 3A12 was Isolated from a Corn Landrace Grown Continuously by Subsistence Farmers since Ancient Times

Strain 3A12 was originally isolated from surface-sterilized seeds of the ancient maize (corn) landrace Chapalote, a type of maize selected by ancient Uto-Aztecan tribes (Wellhausen et al., 1952). Chapalote appears to have been widespread in pre-Columbian times, spreading from coastal Mexico to both the USA and South America, where it gave rise to other landraces (Wellhausen et al., 1952). The plant is still grown today and is widely adapted from the lowlands to highlands of Mexico (Bonavia, 2013). For example, modern Mayo and Tarahumara peoples grind toasted Chapalote to make the beverage *pinole* (Bonavia, 2013). The persistence of this landrace over 1000s of years suggests that it has offered consistent advantages compared to modern crop varieties. In fact, in a study of 28 landraces, Chapalote was shown to have exceptional insect resistance (Arnason et al., 1993). We previously showed that strain 3A12 has fungicidal activity; not only against *S. homoeocarpa*, but a broad spectrum of plant-associated fungi including pathogens (Shehata et al., 2016). Using microscopy and mutagenesis, here we demonstrated that 3A12 kills a plant fungal pathogen by swarming toward it, then attaching and forming microcolonies around it, enabled by biofilm formation. The endophytic behavior of strain 3A12 in creeping bentgrass was confirmed using confocal microscopy (especially shoots). We did not see significant bacterial growth on the outside of the growth media. Nevertheless, we cannot rule out that the bacteria may be exerting some of its antifungal activity outside of the plant.

### The YajQ/c-di-GMP Signaling System

The Tn5 mutant screen revealed that YajQ, a receptor for the secondary messenger c-di-GMP (An et al., 2014), is a critical signaling system that mediates the endophytic mobility-mediated defense of strain 3A12 for its host plant. Severe mutations in *yajQ* resulted in reductions in all virulence phenotypes tested including chitinase activity, biofilm formation, attachment to its fungal target, loss of flagella and reduced swarming motility toward its target, allowing the pathogen to kill its host plant (**Figures 3A,D,F,Q,AB**; Supplementary Table S3). In earlier studies, c-di-GMP has been shown to regulate transcription and can also bind mRNAs and affect gene expression through riboswitches (An et al., 2014). Similar to quorum sensing, some bacteria utilize c-di-GMP signaling to report the surrounding environment and population density (Flemming and Wingender, 2010; Srivastava and Waters, 2012). Chitinase encoding genes were previously found to be upregulated by c-di-GMP (Zogaj et al., 2012). Furthermore, binding of c-di-GMP resulted in transcriptional regulation of anti-pathogen genes required for biofilm formation, adhesion, motility and synthesis of bacterial virulence factors (Landini et al., 2010; Srivastava and Waters, 2012; Römling et al., 2013; An et al., 2014; Chen et al., 2015; Fazli et al., 2015; Steinberg and Kolodkin-Gal, 2015). Here, for the first time, to the best of our knowledge, the YajQ/c-di-GMP signaling system has been shown to underlie a mutualistic endophyte-host interaction of critical importance to its host plant.

### Other Genes Required for the Endophytic Mobility-Mediated Defense System

Earlier studies found that YajQ can regulate or be regulated by other genes [tRNA synthetase and fatty acid desaturase (FAD)] predicted here to be required for anti-fungal activities (Saveanu et al., 2002; Suppiger et al., 2013). These observations suggest that the YajQ/c-di-GMP signaling system may act in concert with some of the other genes identified in our mutant screen to enable mobility-mediated defense.

#### Fatty Acid Desaturase (fad-1C1::Tn5, fad-2C12::Tn5; EC 1.14.19.1); Delta-9 Fatty Acid Desaturase (EC 1.14.19.1)

A FAD creates double bonds in a fatty acid chain. This study identified a FAD as required for anti-fungal activity. Previous studies have demonstrated the role of unsaturated fatty acids in quorum sensing (Kalia et al., 2013), in cross talk signaling for biofilm formation (Suppiger et al., 2013), as structural components of biofilm lipids (Vu et al., 2009), and FAD genes are also components of operons that encode anti-fungal agents (Fritsche et al., 2014; Ye et al., 2014). Interestingly, as already alluded to, previous studies have shown that c-di-GMP is a downstream regulator of fatty acid signaling in the Bcc (Suppiger et al., 2013). Both FAD mutants identified in this study showed decreased biofilm formation and motility and partial loss of flagella (**Figures 3A,D,G,H,R,S**; Supplementary Table S3) as well as reduced attachment and microcolony formation at its fungal target (**Figures 3AC,AD**).

#### Lysine-tRNA Synthetase Like Gene (lys-1B6::Tn5, lys-1C3::Tn5, lys-2D1::Tn5; E.C. 6.1.1.6)

The protein encoded by this gene was found to contain a region with similarity to a conserved domain from lysine-tRNA synthetases responsible for attachment of lysine to tRNA. In a previous study, a c-di-GMP receptor (ortholog to YajQ, above) was shown to interact directly with tRNA (Suppiger et al., 2013) which would suggest that this tRNA synthetase like protein may be part of the YajQ regulon, a hypothesis that will require further testing. Among alternative hypotheses are that this gene may be coding for a nucleotide antibiotic similar to mupirocin (from *Pseudomonas fluorescens*), agrocin 84 (from *Agrobacterium radiobacter*), or phosmidosine (from a *Streptomyces* sp.) (Phillips et al., 1993), with the latter shown to have antifungal activity (Sekine et al., 2001). These molecules act through competitive inhibition of specific tRNA synthetases in their pathogen targets (Phillips et al., 1993; Sekine et al., 2001; Kim et al., 2006; Gurney and Thomas, 2011). In this study, the mutant alleles of this gene (*lys*-1B6::Tn5, *lys*-1C3::Tn5, *lys*-2D1::Tn5) showed reductions in only a subset of the virulence phenotypes disrupted in the YajQ mutants. Specifically, the *lys* mutants showed impaired biofilm formation (**Figure 3A**; Supplementary Table S3), varying degrees of loss of flagella and motility in the absence of the fungal pathogen (including no loss; **Figures 3I–K,T–V**; Supplementary Table S3), but no effect on swarming, attachment and microcolony formation at its fungal target (**Figures 3AE,AG**).

#### YbgC(tol) [ybgC(tol)-2B10::Tn5, ybgC(tol)-2C4::Tn5, ybgC(tol)-2C11::Tn5]

Orthologs of YbgC remain relatively uncharacterized since its initial report (Zhuang et al., 2002) and yet they belong to a large group of proteins that share the same protein fold called the Hot Dog fold for its shape (Labonte and Townsend, 2013). YbgC was speculated to be involved in the operations of the cell envelope, consistent with its placement here within the Tol operon. The Tol-pal protein complex anchors the cytoplasmic membrane to the outer membrane in Gram negative bacteria (Godlewska et al., 2009). Tol-pal proteins are involved in transport of virulence factors (Godlewska et al., 2009) and biofilm formation (Dubuisson et al., 2005). Mutations in *tol* in *Escherichia coli* and *Pseudomonas aeruginosa* caused multiple phenotypes, including reduced motility, adhesion, virulence and pathogenesis (Lo Sciuto et al., 2014; Morgan et al., 2014; Santos et al., 2014). Consistent with the literature, here all the *ybgC(tol)* mutants showed loss of flagella and reduced biofilm formation (**Figures 3A,W–Y**; Supplementary Table S3), and altered attachment and colony formation at the target fungus (**Figures 3AH–AJ**). Only one mutant [*ybgC(tol)*-2C4::Tn5] showed reduced swarming motility while the other two mutants [*ybgC(tol)-*2B10::Tn5 and *ybgC(tol)-*2C11::Tn5] were not affected in motility (**Figures 3L–N**).

#### Arginine Decarboxylase/Ornithine Decarboxylase/Lysine Decarboxylase (ADC/ODC/LDC, adc-2D2::Tn5; ADC: E.C. 4.1.1.19; ODC: 4.1.1.17; LDC: 4.1.1.18)

These closely related enzymes belong to the same family of pyridoxal phosphate (PLP)-dependent aspartate aminotransferases, wherein LDC converts lysine to cadaverine, ADC is responsible for the enzymatic conversion of arginine to agmatine, and ODC converts ornithine to putrescine (Brown et al., 1990; Castagnolo et al., 2011). Arginine can be converted to ornithine by arginase. In the context of the other mutants identified in this study, arginine was previously found to increase c-di-GMP levels and perhaps via this pathway, this amino acid was observed to promote biofilm formation (Chen et al., 2013) and alter swarming (Sturgill and Rather, 2004; Bernier et al., 2011; Armbruster et al., 2013, 2014). Consistent with the literature, in this study, the ADC/ODC mutant caused reduced biofilm formation and loss of flagella, however, the swarming motility was not affected (**Figures 3A,O,Z**; Supplementary Table S3). This mutant showed altered attachment and colony formation at the target fungus (**Figure 3AK**). LDC and the above noted lysine tRNA synthetase like gene may be connected since both are predicted to share lysine as a substrate. Furthermore, arginine uptake across the cytoplasmic membrane is facilitated by the Tol-Pal system (Llamas et al., 2003), potentially further suggestive of an interactive anti-fungal network operating in 3A12. Alternatively, ADC/ODC/LDC may be playing a more active role in the antifungal activity: agmatine derivatives, hydroxycinnamoylagmatines, are the precursors for hordatines, which are antifungal agents found in barley seedlings (Burhenne et al., 2003). Feruloylagmatine is another agmatine derivative with antifungal activity which is found in winter wheat (Jin and Yoshida, 2000). The products of LDC, cadaverine and its derivatives, have been implicated in various processes including as antifungal metabolites (Brown et al., 1990; Niemi et al., 2002).

#### Other Candidate Anti-fungal Genes

Bioinformatic-based mining of the genome sequence of endophyte 3A12 (Ettinger et al., 2015) revealed the presence of other candidate anti-fungal genes including genes predicted to encode known fungicides and antimicrobial compounds, specifically phenazine(s) (Chin-A-Woeng et al., 2003; Pierson and Pierson, 2010), colicin(s) (Gérard et al., 2005) and chitinase(s), as noted above (**Supplementary Figure S2A**; Supplementary Table S3). Phenazines are nitrogen containing aromatic compounds which are known to have broad spectrum anti-pathogen and anti-fungal activities (Chin-A-Woeng et al., 2003; Pierson and Pierson, 2010) and promote induced systemic resistance (ISR) in plants (De Vleesschauwer et al., 2006). Colicin V is a small peptide antibiotic, a type of bacteriocin, that exerts its antibacterial activities by disrupting the cell membrane leading to loss of membrane potential (Gérard et al., 2005). Chitinase is the enzyme that hydrolyzes chitin, a β-1,4- linked polymer of *N*-acetyl D-glucosamine (Dahiya et al., 2005). Aside from its direct hydrolytic activity of fungal cell walls, chitinase-based liberation of *N*-acetyl glucosamine induces plant defense mechanisms (Gohel et al., 2006) and is involved in biofilm formation (Zogaj et al., 2012). Strain 3A12 was found to display chitinase activity, which was induced by the fungal pathogen (**Supplementary Figure S2B,C**), and this activity was disrupted in the *yajQ* mutant, as noted above (Supplementary Table S3).

## Conclusion

**FIGURE 4:**
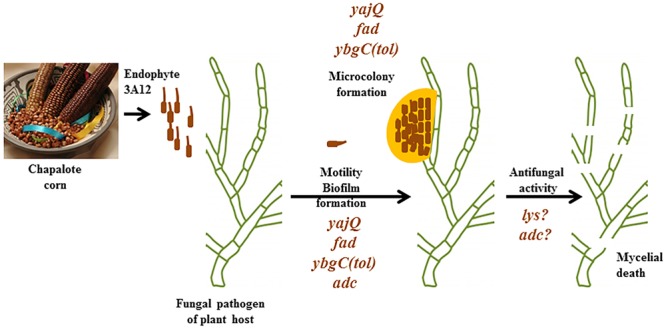
**Summary of how endophyte 3A12 confers mobility-based defense for its host plant and the genes required.** Bacterial endophyte strain 3A12 originates from the ancient Meso-American corn landrace Chapalote and can combat various crop pathogens. Following pathogen sensing, the endophyte swarms toward its target, a fungal pathogen of its host plant, where it forms microcolonies around the fungal pathogen, likely mediated by biofilm, after which the endophyte kills fungal hyphae. Mutant analysis suggests the genes that may be involved in each of these steps. Picture of Chapalote corn courtesy of CIMMYT (under the Creative Commons BY-NC-SA 2.0 License).

Our results show that Chapalote, a crop landrace grown continuously by subsistence farmers since ancient times in the Meso-Americas, possesses a remarkable endophytic microbe. This endophyte has the ability to recognize, then swarm toward a pathogen of its host plant, attach, form microcolonies and finally kill the pathogen (**Figure 4**). Our study demonstrates that the YajQ/c-di-GMP signaling system is critically important for this mobility-mediated defense system, along with a potential network of other endophytic genes. The ancient crops of the world’s indigenous peoples may represent a new frontier in our understanding of plant–microbe mutualistic interactions.

## Author Contributions

HS helped to design the study, carried out all experiments, performed the analyses, and wrote the manuscript. CE and JE conducted genome sequencing and annotation for strain 3A12. MR helped to design the study and edited the manuscript. All authors read and approved the final manuscript.

## Conflict of Interest Statement

The authors declare that the research was conducted in the absence of any commercial or financial relationships that could be construed as a potential conflict of interest.

## Supplementary Material

FIGURE S1 **FIGURE S1 | Genetic complementation of candidate 3A12 mutants with predicted wild type gene fragments, using *in vitro* antifungal assays.** Shown are representative images of inhibition zones of pathogen *S. homoeocarpa* on agar following co-inoculation with **(A)** wild type strain 3A12, **(B,D,F,H)** mutant strains, **(C,E,G,I)** mutant strains expressing gene fragments from the wild type.

FIGURE S2 **FIGURE S2 | Other candidate anti-fungal genes as predicted from the genome sequence of strain 3A12. (A)** List of candidate genes with their position(s) in the genome of strain 3A12, along with their GenBank BLASTN search predictions and functions. The letter S refers to the scaffold number from the published draft genome sequence of strain 3A12 (Ettinger et al., 2015). **(B)** Chitinase assay of strain 3A12 using two different enzyme substrates. **(C)** Chitinase assay of strain 3A12 cultured alone or in the presence of *S. homoeocarpa* using 4-nitrophenyl *N*-acetyl-β-D-glucosaminide as the enzyme substrate. Error bars represent the standard error of the mean. The asterisks indicates a significant increase in chitinase activity when the fungal pathogen was added to the strain 3A12 culture.

FIGURE S3 **FIGURE S3 | Localization of GFP-tagged strain 3A12 to the intercellular spaces and/or vasculature of creeping bentgrass shoots using confocal microscopy.** The image was taken 1 week after the strain was coated onto seeds.
