# Supplementary figures and images for: Genes Required for the Anti-fungal Activity of a Bacterial Endophyte Isolated from a Corn Landrace Grown Continuously by Subsistence Farmers Since 1000 BC
Source: Front Microbiol. 2016 Oct 4;7:1548. doi: 10.3389/fmicb.2016.01548 (PMC5047915; doi:10.3389/fmicb.2016.01548)

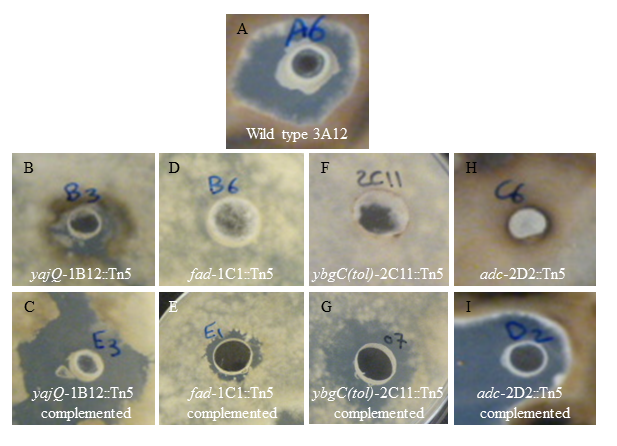

Supplement: FIGURE S1 — | Genetic complementation of candidate 3A12 mutants with predicted wild type gene fragments, using in vitro antifungal assays. Shown are representative images of inhibition zones of pathogen S. homoeocarpa on agar following co-inoculation with (A) wild type strain 3A12, (B,D,F,H) mutant strains, (C,E,G,I) mutant strains expressing gene fragments from the wild type. [file Image_1.tif]

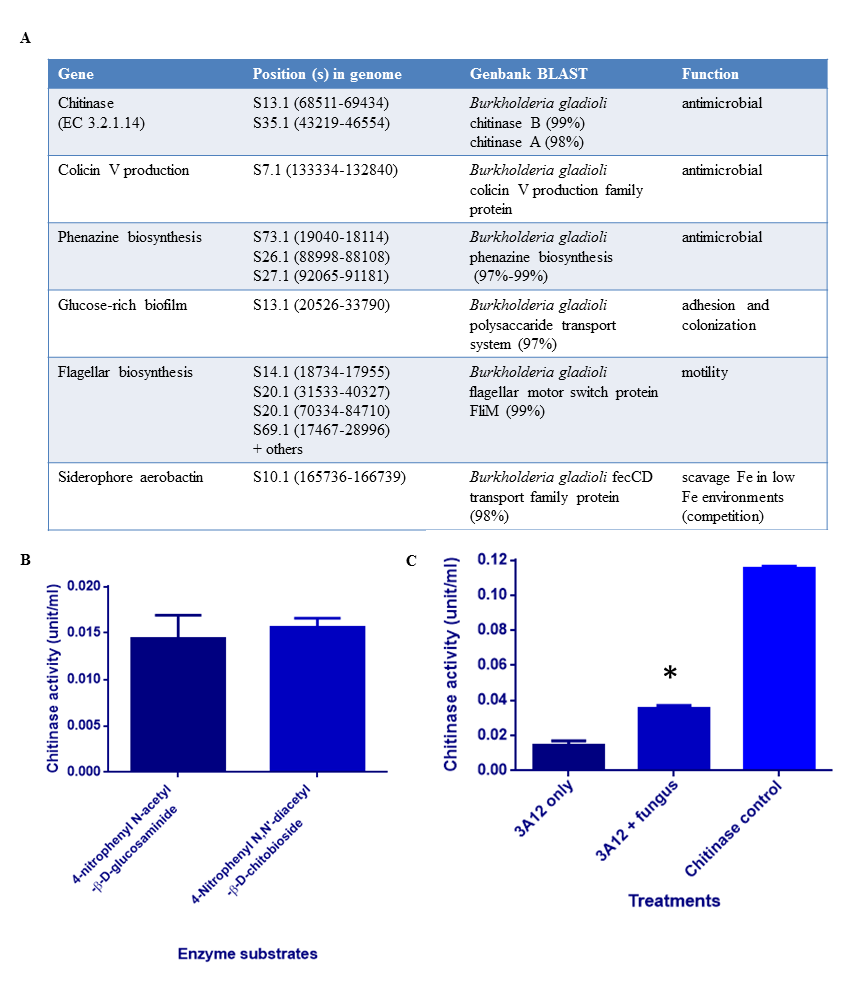

Supplement: FIGURE S2 — | Other candidate anti-fungal genes as predicted from the genome sequence of strain 3A12. (A) List of candidate genes with their position(s) in the genome of strain 3A12, along with their GenBank BLASTN search predictions and functions. The letter S refers to the scaffold number from the published draft genome sequence of strain 3A12 (Ettinger et al., 2015). (B) Chitinase assay of strain 3A12 using two different enzyme substrates. (C) Chitinase assay of strain 3A12 cultured alone or in the presence of S. homoeocarpa using 4-nitrophenyl N-acetyl-β-D-glucosaminide as the enzyme substrate. Error bars represent the standard error of the mean. The asterisks indicates a significant increase in chitinase activity when the fungal pathogen was added to the strain 3A12 culture. [file Image_2.tif]

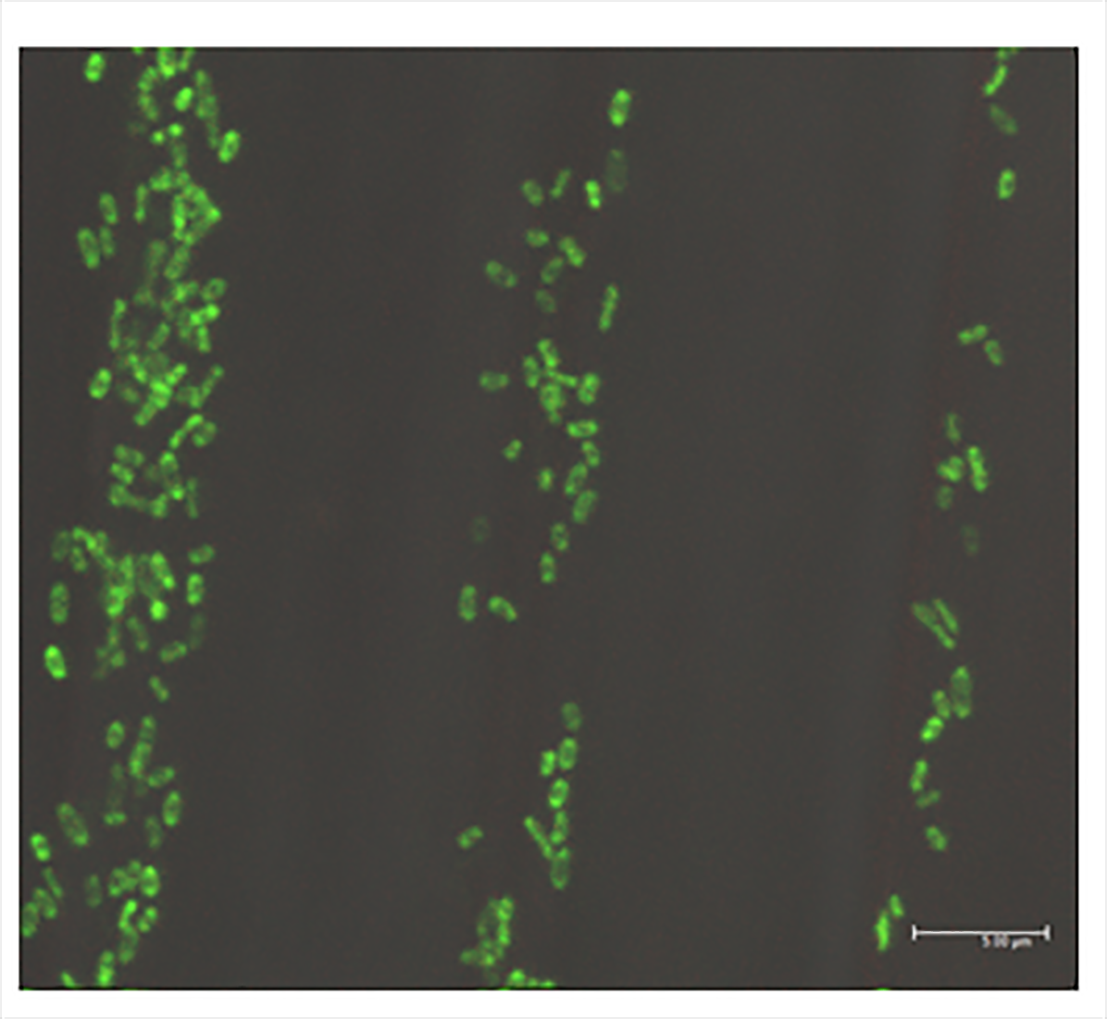

Supplement: FIGURE S3 — | Localization of GFP-tagged strain 3A12 to the intercellular spaces and/or vasculature of creeping bentgrass shoots using confocal microscopy. The image was taken 1 week after the strain was coated onto seeds. [file Image_3.tif]
