# Supplementary material for: Genes Required for the Anti-fungal Activity of a Bacterial Endophyte Isolated from a Corn Landrace Grown Continuously by Subsistence Farmers Since 1000 BC
Source: Front Microbiol. 2016 Oct 4;7:1548. doi: 10.3389/fmicb.2016.01548 (PMC5047915; doi:10.3389/fmicb.2016.01548)
Supplement: Supplementary file 4 [file Table_1.DOCX]

**Table S1:** Sequences of candidate anti-fungal genes from strain 3A12 flanking Tn5-insertion mutants

| **Mutant name** | **Mutant gene sequence** |
| --- | --- |
| m1B6 | GTGTTGCTGCCGCAGCATGAGTTCGCGCGGCTCGACTCGTACTGCGACGAACGCGGCTTCAAGAAAAGTACGCTGATTGCCCGACTGATTCGAGACTACCTCGACAGCGAGCATTTTCCAAACCAAGAAGTACTCCCGCTTGAGGTTGTCACGCCGAGTGTGTCGGGTGCGCCAAGCGACTCGCCCAAGGGGTGA |
| m1B12 | ATGCCATCGTTCGACGTCGTTTCCGAAGCGAACATGATCGAAGTGAAGAATGCCGTCGAGCAGTCGAACAAGGAAATTTCGACGCGCTTCGACTTCAAGGGCTCCGACGCGCGCGTCGAGCACAAGGAACAGGAGCTGACCCTGTTCGCCGACGACGACTTCAAGCTCGGCCAGGTCAAGGACGTGCTGATCGGCAAGATGGCCAAGCGCAATGTCGACGTGCGTTTCCTCGACTACGGCAAGGTCGACAAGATCGGCGGCGACAAGCTCAAGCAGGTCGTCACGATCAAGAAGGGCGTGACGGGCGACCTGGCCAAGCGCGTGGTGCGTACCGTCAAGGACAGCAAGATCAAGGTGCAGGCCAGCATCCAGGGCGACGCGGTGCGCGTGTCGGGCACCAAGCGCGACGACCTGCAGAGCGTGATCGCGCTGCTGCGCAAGGAAGTCGCCGACACGCCGCTGGACTTCAACAACTTCCGCGACTGA |
| m1C1 | GTGCTCGACTTCCTGGCCCACGGGCTGCTGCATTTCTCGTGGTGGCAGATCGTTCTGGCCACGCTTGTGGCCACGCACGTCACGATCGTCTCCGTCACCATCTATCTCCACCGCTGTCAGGCGCATCGCGCGCTCGACCTGCATCCGGCCGTCAGCCACTTCTTCCGGCTGTGGCTGTGGATGTCCACCGGCATGCTGACCGGCCAGTGGGCGGCCATCCATCGCAAGCACCACGCCAAGTGCGAGACCGAAGAGGATCCGCACAGCCCGCAGACGCGCGGCATCTGGAAGGTGCTGCTCGAAGGCGCCGAGCTCTATCGCGCCGAAGCCAAGAACGAGGAAACGCTGCGCAAGTTCAGCCACGGCACGCCGAACGACTGGATCGAGCGCAACGTCTACTCGAAGTACACGATCCTCGGCGTGAGCCTGATGATGGTGATCGACGTCGCGCTGTTCGGCATCGTCGGCCTGTCGGTCTGGGCCGTGCAGATGATCTGGATCCCGTTCTGGGCGGCCGGCGTGGTCAACGGCCTCGCGCACTTCTGGGGCTACCGCAACTTCAACTCGGCCGATGCGAGCACGAACCTGATTCCCTGGGGCATCGTGATCGGCGGCGAAGAAATGCACAACAACCACCACACCTTCGCCACCTCGGCGAAGTTCTCGAACAAGTGGTACGAGTTCGACATCGGCTGGATGTACATCCGCATCCTGTCGGCGTTCAAGCTCGCCAAGGTCAAGAAGATCGCGCCCACGCCGCGCCTGGTGGCACGCAAGGCCGTGGTCGACCAGGAAACGCTGCAGGCCGTGCTGTCGAACCGCTACGAAGTGATGGCGAACTACGGCAAGGCACTGAAGCGCGCTTACCGCCAGGAGCTCGCGCACCTGAAGGAGCTTGGTTCGAGCGAGAAGTACCAACTGCTGCGCGGCGCCCGTTCCTGGTTCCACAAGGACGAAGAGGGCCTGAACGAGCCGCAGAAGCGCCTGCTGCCCGAGATCTTCGCCAACAGCCAGAAGATGCATACCTACTTCCAGCTGCGCCAGGACCTGGCCTCGATGTGGGACCGCTCGAACGCTTCGCGCGAACAGCTGCTGGCCCAATTGCAGGACTGGTGCCATCGTGCCGAACAAAGCGGCATCAAGGCGCTGCAGGAGTTCGCGACGCGTCTGCGCCGCTACGCCTGA |
| m1C3 | GTGTTGCTGCCGCAGCATGAGTTCGCGCGGCTCGACTCGTACTGCGACGAACGCGGCTTCAAGAAAAGTACGCTGATTGCCCGACTGATTCGAGACTACCTCGACAGCGAGCATTTTCCAAACCAAGAAGTACTCCCGCTTGAGGTTGTCACGCCGAGTGTGTCGGGTGCGCCAAGCGACTCGCCCAAGGGGTGA |
| 2B10 | ATGCGCGCCATGACCGAACCCGAACCTCCGCCCGAAGCCGTGCCCTCGTTTCGCTGGCCGGTTCGCGTGTACTACGAGGACACCGATGCCGGCGGCATCGTGTACTACGCGAACTACCTGCGCTTCTTCGAGCGGGCCCGCACCGAATGGCTGCGGGCCTGCGGCCTCGACCAACGGCGCCTGGCCGCCGAGACCGGCGCGATGTTCGTGGTGCGCGACACGGCGATCGACTACCGGGCGCCGGCGCGGCTGGACGATCCGTTGACGATCGTCAGCCGGATCGAACGTTTCGGGCGCGCCTCGCTGATCTTCGTGCAGGAAGCCTGGCTCGGCGACCTGCTGCTGGTGTCGGGGCGTACCGGCATCGGCTGGGTCGACCGCGAAACGCTGCGGCCGACCGGCATTCCGCCCTCGGTCCGCGATGCACTGGAACGGGGACCGGTACCAAGCGTGTCAACGGCCGAGGCCTGA |
| m2C4 | ATGCGCGCCATGACCGAACCCGAACCTCCGCCCGAAGCCGTGCCCTCGTTTCGCTGGCCGGTTCGCGTGTACTACGAGGACACCGATGCCGGCGGCATCGTGTACTACGCGAACTACCTGCGCTTCTTCGAGCGGGCCCGCACCGAATGGCTGCGGGCCTGCGGCCTCGACCAACGGCGCCTGGCCGCCGAGACCGGCGCGATGTTCGTGGTGCGCGACACGGCGATCGACTACCGGGCGCCGGCGCGGCTGGACGATCCGTTGACGATCGTCAGCCGGATCGAACGTTTCGGGCGCGCCTCGCTGATCTTCGTGCAGGAAGCCTGGCTCGGCGACCTGCTGCTGGTGTCGGGGCGTACCGGCATCGGCTGGGTCGACCGCGAAACGCTGCGGCCGACCGGCATTCCGCCCTCGGTCCGCGATGCACTGGAACGGGGACCGGTACCAAGCGTGTCAACGGCCGAGGCCTGA |
| m2C11 | ATGCGCGCCATGACCGAACCCGAACCTCCGCCCGAAGCCGTGCCCTCGTTTCGCTGGCCGGTTCGCGTGTACTACGAGGACACCGATGCCGGCGGCATCGTGTACTACGCGAACTACCTGCGCTTCTTCGAGCGGGCCCGCACCGAATGGCTGCGGGCCTGCGGCCTCGACCAACGGCGCCTGGCCGCCGAGACCGGCGCGATGTTCGTGGTGCGCGACACGGCGATCGACTACCGGGCGCCGGCGCGGCTGGACGATCCGTTGACGATCGTCAGCCGGATCGAACGTTTCGGGCGCGCCTCGCTGATCTTCGTGCAGGAAGCCTGGCTCGGCGACCTGCTGCTGGTGTCGGGGCGTACCGGCATCGGCTGGGTCGACCGCGAAACGCTGCGGCCGACCGGCATTCCGCCCTCGGTCCGCGATGCACTGGAACGGGGACCGGTACCAAGCGTGTCAACGGCCGAGGCCTGA |
| m2C12 | GTGCTCGACTTCCTGGCCCACGGGCTGCTGCATTTCTCGTGGTGGCAGATCGTTCTGGCCACGCTTGTGGCCACGCACGTCACGATCGTCTCCGTCACCATCTATCTCCACCGCTGTCAGGCGCATCGCGCGCTCGACCTGCATCCGGCCGTCAGCCACTTCTTCCGGCTGTGGCTGTGGATGTCCACCGGCATGCTGACCGGCCAGTGGGCGGCCATCCATCGCAAGCACCACGCCAAGTGCGAGACCGAAGAGGATCCGCACAGCCCGCAGACGCGCGGCATCTGGAAGGTGCTGCTCGAAGGCGCCGAGCTCTATCGCGCCGAAGCCAAGAACGAGGAAACGCTGCGCAAGTTCAGCCACGGCACGCCGAACGACTGGATCGAGCGCAACGTCTACTCGAAGTACACGATCCTCGGCGTGAGCCTGATGATGGTGATCGACGTCGCGCTGTTCGGCATCGTCGGCCTGTCGGTCTGGGCCGTGCAGATGATCTGGATCCCGTTCTGGGCGGCCGGCGTGGTCAACGGCCTCGCGCACTTCTGGGGCTACCGCAACTTCAACTCGGCCGATGCGAGCACGAACCTGATTCCCTGGGGCATCGTGATCGGCGGCGAAGAAATGCACAACAACCACCACACCTTCGCCACCTCGGCGAAGTTCTCGAACAAGTGGTACGAGTTCGACATCGGCTGGATGTACATCCGCATCCTGTCGGCGTTCAAGCTCGCCAAGGTCAAGAAGATCGCGCCCACGCCGCGCCTGGTGGCACGCAAGGCCGTGGTCGACCAGGAAACGCTGCAGGCCGTGCTGTCGAACCGCTACGAAGTGATGGCGAACTACGGCAAGGCACTGAAGCGCGCTTACCGCCAGGAGCTCGCGCACCTGAAGGAGCTTGGTTCGAGCGAGAAGTACCAACTGCTGCGCGGCGCCCGTTCCTGGTTCCACAAGGACGAAGAGGGCCTGAACGAGCCGCAGAAGCGCCTGCTGCCCGAGATCTTCGCCAACAGCCAGAAGATGCATACCTACTTCCAGCTGCGCCAGGACCTGGCCTCGATGTGGGACCGCTCGAACGCTTCGCGCGAACAGCTGCTGGCCCAATTGCAGGACTGGTGCCATCGTGCCGAACAAAGCGGCATCAAGGCGCTGCAGGAGTTCGCGACGCGTCTGCGCCGCTACGCCTGA |
| m2D1 | GTGTTGCTGCCGCAGCATGAGTTCGCGCGGCTCGACTCGTACTGCGACGAACGCGGCTTCAAGAAAAGTACGCTGATTGCCCGACTGATTCGAGACTACCTCGACAGCGAGCATTTTCCAAACCAAGAAGTACTCCCGCTTGAGGTTGTCACGCCGAGTGTGTCGGGTGCGCCAAGCGACTCGCCCAAGGGGTGA |
| m2D2 | ATGAAGTTTCGTTTTCCCGTCGTCATCATCGACGAAGATTTCCGCTCCGAGAACATCTCGGGCTCCGGCATCCGGGCACTTGCCGAAGCGATCGAGAAGGAAGGGGTGGAAGTCCTCGGCCTGACGAGCTACGGCGATCTGACCTCGTTCGCGCAGCAGTCGAGCCGCGCGTCGTGCTTCATCCTGTCGATCGACGATGACGAGCTGATGCTCGGGGAAACCGGCCCGGACGGCGAACTGCCCGAACTGGCCACCGCCATCCTGGAACTGCGCGCCTTCGTGACCGAAGTGCGCCGCCGCAACGCCGACATCCCGATCTTCCTGTACGGCGAGACGCGCACCTCGCGCCATATTCCGAACGACGTGCTGCGCGAGCTGCACGGCTTCATCCACATGTTCGAGGACACGCCGGAGTTCGTGGCGCGCCACATCATCCGCGAGACCAAGGTCTACCTCGATTCGCTGGCGCCGCCGTTCTTCAAGGAGCTGGTCAAGTACGCCGACGAGGGCTCCTATTCGTGGCACTGCCCGGGGCACTCGGGCGGGGTGGCCTTCCTGAAGAACCCGCTCGGCCAGATGTTCCACCAGTTCTTCGGCGAGAACATGCTGCGCGCCGACGTCTGCAACGCCGTCGACGAACTCGGCCAGCTGCTCGACCATACCGGCCCGGTGGCCGCCTCGGAGCGCAATGCCGCGCGTATCTTCAGCGCCGACCACCTGTTCTTCGTCACCAACGGCACCTCGACCTCGAACAAGATCGTCTGGCACGCCAACGTGGCGCCGGGCGACATCGTTCTGGTCGACCGCAACTGCCACAAGTCGATCCTGCACGCGATCACCATGACGCATGCGATTCCCGTGTTCCTGACGCCGACGCGCAACCACTTCGGCATCATCGGGCCGATCCCGCGCGACGAGTTCAAGCCGGAGAACATCCGCAAGAAGATCGAGGCGAACCCGTTCGCCCGCGAGGCGCTGCAGAAGAACCCGAACGCCAAGCCGCGGATCCTGACGATCACCCAGAGCACCTACGACGGCGTGATCTACAACGTCGAGCACATCAAGGACCTGCTGGGCGACCTGCTCGACACGCTGCACTTCGACGAGGCCTGGCTGCCGCACGCGGAATTCCACGAGTTCTACCGCGACATGCACGCGATCGGCGCGGGCCGACCGCGCACCGGCTCGCTGGTGTTCGCCACGCACTCCACCCACAAGCTGCTGGCCGGCATCTCGCAGGCCTCGCAGATCGTGGTGCAGGACTCGGAGAACCGCACCTTCGACAAGCACCGCTTCAACGAGGCCTACCTGATGCATACCTCGACCAGCCCGCAGTACGCCATCATCGCCTCGTGCGACGTGGCCGCCGCGATGATGGAGCCGCCCGGCGGCACCGCGCTGGTCGAGGAGTCGATCGCCGAGGCGATCGAGTTCCGCCGCGCGATGCGCAAGGTCGACGCCGAATACGGCGACGACTGGTTCTTCTCGGTGTGGGGCCCGGACACGCTGCCCGAGGAAGGGATCGGCTCGCGCGAGGACTGGATCCTGCGCCCGAACGACCGCTGGCACGGCTTCGGCCCGCTCGCGGAAGGCTTCAACATGCTGGACCCGATCAAGGCCACCATCATCACCCCGGGCCTGGACGTGGACGGCGAGTTCGGCGAGACCGGGATTCCGGCGGCGATCGTCACCAAGTACCTGGCCGAGCACGGCATCATCGTCGAGAAGACGGGCCTCTACTCGTTCTTCATCATGTTCACGATCGGCATCACCAAGGGCCGCTGGAACTCGATGGTCACCGAGCTGCAGCAGTTCAAGGACGATTACGACAACAATCAGCCGCTCTGGCGCGTGCTGCCCGAGTTCGTGGCGCAGTTCCCGATCTACGAGCGCGTCGGCCTGCGCGACCTCTGCACGCAGATCCACGACGTCTACCGCGCCAACGACATCGCGCGCCTGACCACCGAGATGTATCTCTCGGACATGGAGCCGGCGATGAAGGCCTCGGACGCGTTCGCCAAGCTCGCGCACCGCGAGATCGACCGCGTGCCGCTCGACGAGCTGGAAGGGCGCGTCACCAGCATCCTGCTCACGCCGTATCCGCCGGGTATTCCGCTGCTGATCCCGGGCGAGCGCTTCAATGCCACGATCGTCAACTACCTGCGTTTCGCCCGCGATTTCAACGAGCGTTTCCCGGGTTTCCATACCGACGTGCATGGCCTGGTGGCCGAGGAAGTGAACGGGCGCGTCGAGTACTACGTCGACTGCGTGCGCGACTGA |
