# Supplementary material for: Genes Required for the Anti-fungal Activity of a Bacterial Endophyte Isolated from a Corn Landrace Grown Continuously by Subsistence Farmers Since 1000 BC
Source: Front Microbiol. 2016 Oct 4;7:1548. doi: 10.3389/fmicb.2016.01548 (PMC5047915; doi:10.3389/fmicb.2016.01548)
Supplement: Supplementary file 5 [file Table_2.docx]

**Table S2:** Primers used to amplify wild-type gene fragments for the genetic complementation assays.

|  | Primer name | Primer sequence | Product size |
| --- | --- | --- | --- |
| 1 | ADC_F | CCGCGGTCGTTCTTTTTGGA | 2501 bp |
| 2 | ADC_R | GGCCGACCGTCGAGGA |  |
| 3 | YbgC(tol)-F | CATTCGGTTACGAAACGCCG | 525 bp |
| 4 | YbgC(tol)-R | CTCTGTCTAAACGGCGCTCA |  |
| 5 | FAD_F | CCTTGCTGAATTCTGTGCTCG | 1237 bp |
| 6 | FAD_R | GGGGTTTGTGACGTTCCGA |  |
| 7 | YajQ F | CCCGCATGTCTTACAGGGAG | 517 bp |
| 8 | YajQ R | TTCGATCAGTCGCGGAAGTT |  |
| 9 | T7 | GTAATACGACTCACTATAG |  |
| 10 | SP6 | CATTTAGGTGACACTATAG |  |
