# Supplementary material for: Genes Required for the Anti-fungal Activity of a Bacterial Endophyte Isolated from a Corn Landrace Grown Continuously by Subsistence Farmers Since 1000 BC
Source: Front Microbiol. 2016 Oct 4;7:1548. doi: 10.3389/fmicb.2016.01548 (PMC5047915; doi:10.3389/fmicb.2016.01548)
Supplement: Supplementary file 6 [file Table_3.docx]

**Table S3:** Summary of the functions of the candidate anti-fungal genes based on phenotypes of the Tn5 mutants.

| Mutant names | Growth rate^*^ | *In vitro* antifungal^**^ | *In planta*  Antifungal^***^ | Motility^#^ | TEM  % flagella^##^ | Biofilm^###^ | Chitinase activity^####^ |
| --- | --- | --- | --- | --- | --- | --- | --- |
| **WT 3A12** | 1.13±0.12 | 2.63±0.19 | 0/3 | 4.22±0.23 | 40% | 0.32±0.032 | 0.022±0.002 |
| ***yajQ*-1B12::Tn5** | 1.17±0.03 | 1.90±0.15 | 3/3 | 1.18±0.01 | 0% | 0.06±0.019 | 0.009±0.002 |
| ***fad*-1C1::Tn5** | 1.17±0.03 | 0±0 | 3/3 | 3.14±0.05 | 10% | 0.04±0.031 | 0.024±0.001 |
| ***fad*-2C12::Tn5** | 1.17±0.03 | 0±0 | 3/3 | 4.12±0.08 | 30% | 0.02±0.007 | 0.028±0.003 |
| ***lys*-1B6::Tn5** | 1.27±0.09 | 1.57±0.03 | 3/3 | 1.29±0.02 | 0% | 0.03±0.011 | 0.029±0.004 |
| ***lys*-1C3::Tn5** | 1.23±0.07 | 0±0 | 3/3 | 3.23±0.19 | 40% | 0.01±0.003 | 0.025±0.003 |
| ***lys*-2D1::Tn5** | 1.2±0.06 | 0±0 | 2/3 | 4.4±0.11 | 20% | 0.03±0.005 | 0.028±0.004 |
| ***ybgC(tol)*-2C4::Tn5** | 0.97±0.07 | 1.80±0.03 | 2/3 | 1.12±0.02 | 10% | 0.04±0.01 | 0.016±0.002 |
| ***ybgC(tol)*-2C11::Tn5** | 0.93±0.12 | 0±0 | 3/3 | 3.94±0.14 | 10% | 0.01±0.002 | 0.021±0.001 |
| ***ybgC(tol)-*2B10::Tn5** | 1.2±0 | 0±0 | 3/3 | 4.08±0.22 | 0% | 0.04±0.009 | 0.018±0.002 |
| ***adc*-2D2::Tn5** | 0.87±0.03 | 0±0 | 3/3 | 3.95±0.06 | 0% | 0.01±0.005 | 0.018±0.001 |

*** Mean OD _595nm_ following 24 hr of growth ± SEM**

**** Mean diameter of zone of inhibition (cm)** **± SEM**

***** Number of tubes with sick plants out of 3 tubes**

**# Mean colony diameter ± SEM**

**## Percentage of bacterial cells that were observed to have flagella**

**### Mean absorbance A_570nm_ (Crystal violet) ± SEM**

**#### Mean chitinase activity (unit/ml) ± SEM**
