# Supplementary material for: Genes Required for the Anti-fungal Activity of a Bacterial Endophyte Isolated from a Corn Landrace Grown Continuously by Subsistence Farmers Since 1000 BC
Source: Front Microbiol. 2016 Oct 4;7:1548. doi: 10.3389/fmicb.2016.01548 (PMC5047915; doi:10.3389/fmicb.2016.01548)
Supplement: Supplementary file 7 [file Data_Sheet_1.DOCX]

**Supplemental Materials and Methods (Shehata et al.)**

***Testing candidate mutants for loss of antifungal activity on creeping bentgrass***

*Preparation of bacterial inoculum:* Bacteria were allowed to grow overnight in LB medium at 37 ᵒC with shaking at 250 rpm. Cells were collected by centrifugation for 10 min at 4000 rpm, washed twice in 10 mM tris HCl (pH 7), suspended in 10 mM tris HCl (pH 7) to OD_595_=0.5. Five hundred microliters of each bacterial suspension were diluted in 5 ml of 9.3% PVP aqueous solution (P-5288, Sigma, USA) in 15 ml Falcon tubes. These bacteria-PVP mixtures were used to coat turfgrass seeds.

*Surface sterilization of turfgrass seeds:* Creeping bentgrass seeds were surface sterilized by washing for 1 min with 70% ethanol then for 20 min with bleach and finally washed 6 times with sterile, double distilled water.

*Coating of turfgrass seeds:* Surface sterilized creeping bentgrass seeds were added to each bacteria-PVP mixture (5 ml) and coated for 1 h on a low speed rotary shaker.

*Growing turfgrass:* A Phytagel based modified MS medium was used to germinate and grow creeping bentgrass. The MS medium (pH 5.8) consisted of (per L): half-strength modified basal salt MS (M571, Phytotechnology Laboratories, USA), 250 µl of nicotinic acid (1 mg/ml), 500 µl of pyridoxine HCl (0.5 mg/ml), 5 ml of thiamine HCl (100 mg/l), 500 µl of glycine (2 mg/ml), and 2 g Phytagel (P8169, Sigma, USA) in double distilled water. To help solidify Phytagel, 0.166 g/l of CaCl_2_ was added along with 90 mg/l MgSO_4_. Fifteen ml of the sterile MS medium were again aliquoted into sterile 15 cm X 25 mm covered glass tubes (C5916, Sigma, USA), covered by caps (C5791, Sigma, USA) and autoclaved. Per tube, 20 endophyte-coated creeping bentgrass seeds were placed on the media surface. There were 3 replicate tubes per treatment, randomly distributed in the growth chamber. Seeds were allowed to germinate in the dark for 7 days at room temperature then moved to a growth chamber (BTC-60, Enconair, Winnipeg, Canada) and grown under the following conditions: 25˚C constant, 16 h of cool white fluorescent light (Philips F72T8/TL841/HO 65W, 115-145 µmol m^-2^ s^-1^ measured using a Quantum Meter model BQM, Apogee Instruments Inc, Logan, UT, USA). Seeds coated with PVP without any bacteria were used as the negative control.

*Reinoculation of turfgrass:* On the 15th day of turfgrass growth, each glass tube was re-inoculated with 100 µl of bacterial cell suspension (OD_595_=0.5 in 10 mM tris HCl, pH 7) onto seed surfaces. For negative control plants, 100 µl of 10 mM tris HCl (pH 7) were used.

*Inoculation with* *S. homoeocarpa (as applicable):* *S. homoeocarpa* pathogen strains were grown on PDA for 5 days at 28˚C. Fungal inoculation discs from this media were made using a 1 cm wide tube (previously autoclaved) and then one disc was dropped into each tube of turfgrass after 10 days of plant growth. Seeds coated with endophyte 3A12 and inoculated with *S. homoeocarpa,* were used as a positive control. Seeds coated with PVP without any endophytes added, and not inoculated with *S. homoeocarpa,* were used as a negative control. Tubes were recorded for infection intensity 3-5 weeks later.

**GFP-tagging and microscopic imaging of strain 3A12**

*Preparation of competent cells from strain 3A12:* Strain 3A12 was cultured in LB media and incubated overnight at 37˚C with shaking at 250 rpm. Five ml from the overnight culture were used to inoculate 500 ml of LB and incubated at 37˚C with shaking at 250 rpm. When the optical density (OD_595_) reached 0.3-0.4, the flask was chilled on ice. The culture was then centrifuged at 4000 x *g* for 10 min at 4˚C. The pellet was washed twice in 250 ml of ice cold water, centrifuged as above, resuspended in 10 ml of ice cold 10% glycerol, centrifuged and resuspended in 2 ml of ice cold 10% glycerol. The final cell suspension was dispensed as 40 µl aliquots and quick frozen in liquid nitrogen, then stored at -80˚C.

*Electroporation of GFP plasmid into strain 3A12 competent cells:* Competent cells of strain 3A12 were thawed on ice, then mixed with 50 ng of plasmid vector (pDSK-GFPuv) ([Wang et al., 2007](#_ENREF_3)) and transformed by electroporation. Cells were immediately recovered in 1 ml of LB, incubated with shaking at 250 rpm and 37˚C for 1 h, and then 100 µl of the mixture were streaked on LB plates supplemented with 25 µg/ml kanamycin. Plates were incubated at 37˚C overnight. Bacterial colonies were examined under UV to confirm tagging.

*Plant protocols:* The plant growth protocols used were as described above, without infection with *S. homoeocarpa*, with the following additional modifications:

*Coating GFP-tagged 3A12 onto creeping bentgrass seeds:* GFP-tagged strain 3A12 was introduced onto creeping bentgrass seeds as a seed coat (see above). Plants were examined under a Leica Confocal Laser Scanning Microscope SP5 after 1-2 weeks. Excitation was provided using a 488 nm laser and RSP500 beam splitter. Emission was measured between 500-550 nm for GFP and between 624-680 nm for autofluoresence.

*Spray of GFP-transformed 3A12 onto creeping bentgrass plants:* Seeds of creeping bentgrass were allowed to grow on MS media (see above). Following 4 weeks of growth, plants were sprayed with 1 ml/tube of an overnight culture of GFP-tagged 3A12 (OD_595_=1.0) then examined by confocal microscopy as described above.

**Identification of the gene(s) responsible for the antifungal activity**

*Tn5 Mutagenesis:* The EZ-Tn5 <R6Kγori/KAN-2>Tnp Transposome™ Kit was used (TSM08KR, Epicentre, USA). One µl of transposome was electroporated into 40 µl of 3A12 competent cells. Electroporated cells were immediately recovered in 1 ml of LB media, transferred to a 5 ml tube and incubated on a shaker incubator at 37˚C for 1 h at 250 rpm. One hundred microliters of undiluted cells were plated on LB agar plates supplemented with 25 µg/ml kanamycin. Plates were incubated at 37˚C, and colonies were screened for *in vitro* loss of antifungal activity (see below).

*Mutant screen:* Tn5 insertions were screened for loss of the zone of inhibition of *S. homoeocarpa* growth using the dual culture method. Starting 3 days before the mutant screen, *S. homoeocarpa* was cultured in YPD liquid media at 25˚C at 80 rpm. Potato dextrose agar (PDA) was melted, allowed to cool to 50˚C, mixed with the *S. homoeocarpa* culture at a ratio of 1:25 (v/v); the PDA mixed with *S. homoeocarpa* culture was poured into plates and allowed to completely solidify. Holes were created into the agar using a sterile Pasteur pipette, and the resulting agar plugs were removed using a sterilized wire loop. In parallel, endophyte 3A12-Tn5 insertion colonies recovered on LB plates were cultured in liquid LB media overnight at 37˚C with shaking at 250 rpm. The optical densities of the growing Tn5-containing cultures were measured and only those giving optical densities similar to the wild type 3A12 were used (OD_595_= 0.3-0.5). Thirty microliters from each Tn5 insertion culture were applied into the agar holes surrounded by *S. homoeocarpa* using triplicate plates. Plates were incubated at 25˚C for 5 days, and fungal growth inhibition zones were measured and recorded. In total, ~3000 unique Tn5-containing cultures were screened. A Tn5 insertion that consistently displayed no inhibition zone or an inhibition zone smaller than wild type was recorded as a candidate mutant.

*Growth rate of candidate mutants:* To confirm that the candidate mutants did not lose their antifungal activity because of slower growth rates, OD_595nm_ was measured following overnight incubation in LB at 37°C and shaking at 250 rpm, in triplicate.

*In planta confirmation of loss of antifungal activity:* Wild type 3A12 and candidate mutants were applied onto creeping bentgrass as seed coats then inoculated with *S. homoeocarpa* and evaluated for disease symptoms (see above).

*Plasmid rescue and BLAST analysis:* Candidate mutants were cultured in LB liquid media supplemented with 25 µg/ml kanamycin overnight at 37˚C with shaking at 250 rpm. Genomic DNA was extracted using a Bacterial Genomic DNA Isolation Kit (#17900, Norgen Biotek, Canada). Five hundred nanograms to one microgram of DNA was digested using *Bam*HI (#15201023, Invitrogen, USA) at 37˚C for 1 h, then the reaction mixture was purified using illustra GFX PCR DNA and Gel Band Purification Kits (#28-9034-70, GE Healthcare, USA). The purified fragments were self-ligated using ExpressLink™ T4 DNA Ligase (A13726, Invitrogen, USA) for 1 h at room temperature. The reaction mixtures were purified again using illustra GFX PCR DNA and Gel Band Purification Kits. Three microliters of each ligation mixture were electroporated into TransforMax™ EC100D™ pir-116 Electrocompetent *E.coli* (EC6P095H, Epicentre, USA). Electrocompetent *E.coli* cells were recovered in 1 ml of LB and incubated on a shaker at 37˚C and 250 rpm for 1 h. Recovered DH5α cells were plated on LB plates supplemented with 25 µg/ml kanamycin, and plates were incubated at 37˚C. Plasmid DNA was extracted from recovered colonies using a QIAprep Spin Miniprep Kit (#27106, Qiagen, USA) and submitted for sequencing at the University of Guelph Genomics Facility using the following primers (supplied in the kit): Forward Primer, KAN-2 FP-1 (5′ - ACCTACAACAAAGCTCTCATCAACC - 3′), and Reverse Primer, R6KAN-2 RP-1 (5′ - CTACCCTGTGGAACACCTACATCT - 3′). Amplicon nucleotide sequences were analyzed using BLAST analysis against the genome sequence of strain 3A12.

*Genetic complementation assays:* Strain 3A12 was cultured in LB overnight at 37˚C with shaking at 250 rpm. Genomic DNA was extracted from strain 3A12 using the Bacterial Genomic DNA Isolation Kit (17900, Norgen Biotek, Canada) according to the manufacturer’s protocol. Candidate genes were amplified from genomic DNA of wild-type 3A12 using the primers listed in Table S2. The reaction mixture (total volume of 40 µl) contained 20 µl of GoTaq® Green Master Mix (M712C, Promega, USA), 1 μl of 10 μM forward primer and 1 μl of 10 μM reverse primer, 50 ng of DNA and double distilled water up to 40 μl. The PCR products were run on a 1.5% agarose gel in TBE at 140 V for 1 h. The bands were cut from the gel and purified using Illustra GFX PCR DNA and Gel Band Purification Kits (28-9034-70, GE Healthcare, USA). The PCR products were ligated into a cloning vector using the Qiagen PCR Cloning Plus Kit (231222, Qiagen, Germany) and transformed into Qiagen EZ Component Cells. The transformed cells were plated on LB agar supplemented with carbenicillin, X-gal and IPTG, as recommended by the manufacturer, and incubated at 37°C overnight. One of the resulting white colonies was picked for each gene, streaked on LB agar supplemented with carbenicillin, X-gal and IPTG. The ligation was confirmed using primers T7 and SP6 (Table S2). One colony was used to inoculate LB supplemented with carbenicillin and incubated overnight at 37°C with shaking at 250 rpm. Plasmid DNA was extracted using the QIAprep Spin Miniprep Kit (27106, Qiagen, Germany). Competent cells were prepared from each candidate mutant as described above. Extracted plasmids were electroporated into competent cells of the corresponding mutant and then transformed cells were plated onto LB agar supplemented with carbenicillin and incubated at 37°C for 1-2 days. Resulting colonies were tested for antifungal activity as described above using wild type A12 and the corresponding mutant as controls.

**References**

Easom, C., and Clarke, D. (2008). Motility is required for the competitive fitness of entomopathogenic *Photorhabdus luminescens* during insect infection. *BMC Microbiol.* 8**,** 168.

Stepanović, S., Vuković, D., Dakić, I., Savić, B., and Švabić-Vlahović, M. (2000). A modified microtiter-plate test for quantification of staphylococcal biofilm formation. *J. Microbiol. Methods* 40**,** 175-179.

Wang, K., Kang, L., Anand, A., Lazarovits, G., and Mysore, K.S. (2007). Monitoring *in planta* bacterial infection at both cellular and whole-plant levels using the green fluorescent protein variant GFPuv. *New Phytol.* 174**,** 212-223. doi: 10.1111/j.1469-8137.2007.01999.x.
